# A single-cell liver atlas of *Plasmodium vivax* infection

**DOI:** 10.1016/j.chom.2022.03.034

**Published:** 2022-04-19

**Authors:** Liliana Mancio-Silva, Nil Gural, Eliana Real, Marc H. Wadsworth, Vincent L. Butty, Sandra March, Niketa Nerurkar, Travis K. Hughes, Wanlapa Roobsoong, Heather E. Fleming, Charlie A. Whittaker, Stuart S. Levine, Jetsumon Sattabongkot, Alex K. Shalek, Sangeeta N. Bhatia

**Affiliations:** 1 Institute for Medical Engineering and Science, Massachusetts Institute of Technology (MIT), Cambridge, MA, 02139 USA; 2 David H. Koch Institute for Integrative Cancer Research, MIT, Cambridge, MA, 02139 USA; 3 Institut Pasteur, Université Paris Cité, Inserm U1201, CNRS EMR9195, Unité de Biologie des Interactions Hôte-Parasite, F-75015 Paris, France; 4 Department of Chemistry, MIT, Cambridge, MA 02139, USA; 5 BioMicro Center, MIT, Cambridge, MA 02139, USA; 6 Mahidol Vivax Research Unit, Faculty of Tropical Medicine Mahidol University, Bangkok 10400, Thailand; 7 Ragon Institute of Massachusetts General Hospital, MIT, and Harvard, Cambridge, MA 02139, USA; 8 Broad Institute of MIT and Harvard, Cambridge, MA, 02139, USA; 9 Howard Hughes Medical Institute, Chevy Chase, MD, 20815 USA; 10 Equal contributions; 11 Lead contact

## Abstract

Malaria-causing *Plasmodium vivax* parasites can linger in the human liver for weeks to years, and reactivate to cause recurrent blood-stage infection. Although an important target for malaria eradication, little is known about the molecular features of replicative and non-replicative intracellular liver-stage parasites and their host cell dependence. Here, we leverage a bioengineered human microliver platform to culture patient-derived *P. vivax* parasites for transcriptional profiling. Coupling enrichment strategies with bulk and single-cell analyses, we capture both parasite and host transcripts in individual hepatocytes throughout the course of infection. We define host- and state-dependent transcriptional signatures and identify unappreciated populations of replicative and non-replicative parasites that share features with sexual transmissive forms. We find that infection suppresses transcription of key hepatocyte function genes and elicits an anti-parasite innate immune response. Our work provides a foundation for understanding host-parasite interactions and reveals insights into the biology of *P. vivax* dormancy and transmission.

## INTRODUCTION

*Plasmodium* parasite, the causative pathogen of malaria, has a complex life cycle that spans multiple hosts. Disease transmission is initiated upon bite of an infected *Anopheles* mosquito, which deposits infective parasites, called sporozoites, into the human host. Sporozoites travel to the liver, invade hepatocytes and replicate, forming thousands of new parasites called merozoites, which eventually break out into the blood stream, cyclically invading erythrocytes and initiating clinical symptoms. Parasite transmission back to the mosquito is ensured by the development of sexual gametocyte forms during the asexual erythrocytic cycle that are taken up upon bite to restart the life cycle in the insect host. Uniquely, in the case of *P. vivax*, a subset of liver-stage parasites develops into dormant forms called hypnozoites, which can re-activate weeks to years after initial infection to cause relapsing disease. Thus, the liver-stage, which is obligate yet clinically silent and includes relapse-causing hypnozoites, presents a unique opportunity for malaria intervention before onset of symptoms. However, our knowledge of liver-stage malaria, and the response of its hepatocyte host to infection is sparse due to difficult access to the parasite and lack of suitable human liver models. To date, much of our historical knowledge has been based on liver biopsies of infected patients, making it challenging to perform mechanistic studies on liver-stage forms, especially the quiescent hypnozoites. Transcriptomic studies hold promise for unveiling mechanistic insight into liver-stage *P. vivax* relapsing biology, but the low infection rate and the reduced quantity of parasite transcripts in a transcriptionally active host cell environment has made it difficult to perform these studies.

We recently provided some of the first insights into the transcriptional features of *P. vivax* liver-stages by leveraging an *in vitro* primary human liver platform (MPCC, micropatterned co-cultures) that recapitulates key aspects of *P. vivax* liver-stage biology, including establishment of persistent dormant forms, growing schizonts, merozoite release, and subsequent infection of overlaid erythrocytes. Our previous work revealed reduced transcriptional activity in hypnozoite-enriched samples, specifically, suppressed transcripts for functions related to cell division and invasion machinery, consistent with a quiescent state (Gural et al., 2018). However, the single time-point bulk sequencing used in the previous study prevented us from capturing the inherent heterogeneity of the distinct liver-stage parasite forms as well as host-target and bystander cell responses. Achieving a deeper understanding of pathogen-host interactions has the potential to provide insight into mechanisms that could be leveraged to treat or prevent infection, as suggested by innate interferon responses to infection by rodent malaria parasites (Liehl et al., 2013) and a number of viruses (Schneider et al., 2014). However, a closer look into infection-specific host responses and potential protective responses in uninfected hepatocytes requires single-cell resolution. Recently, diverse single-cell technologies have revealed stage-specific transcriptional signatures in mosquito (Real et al., 2021), blood and gametocyte (Poran et al., 2017; Walzer et al., 2018) stages from non-relapsing human parasites, and the entire life cycle of rodent parasites (Howick et al., 2019), all of which can be easily cultured and propagated in laboratories. For *P. vivax*, single-cell transcriptomic studies have been conducted in blood-stages collected from infected monkeys (Sà et al., 2020), but analysis of liver infection has not been performed to date.

Here, we present a comprehensive view of the liver-stage transcriptomes of a human-infecting malaria parasite and its host cells at single-cell resolution. To achieve this goal, we coupled MPCC with Seq-Well, a recently developed low-cost and portable single-cell platform that does not require fluorescent labeling and is compatible with use of samples collected in endemic settings (Gierahn et al., 2017). With this combined platform, we define distinct signatures between early, dormant, mid, and late-stage parasites, and identify sub-populations of sexually committed forms in the liver, previously thought to appear only during erythrocytic infection. We interrogate pathogen-host interactions and describe dynamic innate immune responses in infected and uninfected bystander cells, suggesting a likely mechanism for endogenous protection from secondary infections. We report cytokine- and stage-dependent anti-parasite activity by induction of interferon signaling and validate host factors involved in cell autonomous innate defense. Together, the data presented here unveils the *P. vivax* liver-stage development with unprecedented resolution and offer insights into the host pathways that might impact their unique biology.

## RESULTS

### Profiling liver-stage *P. vivax* infection by pairing targeted sequencing with single-cell analysis

**Figure 1.**
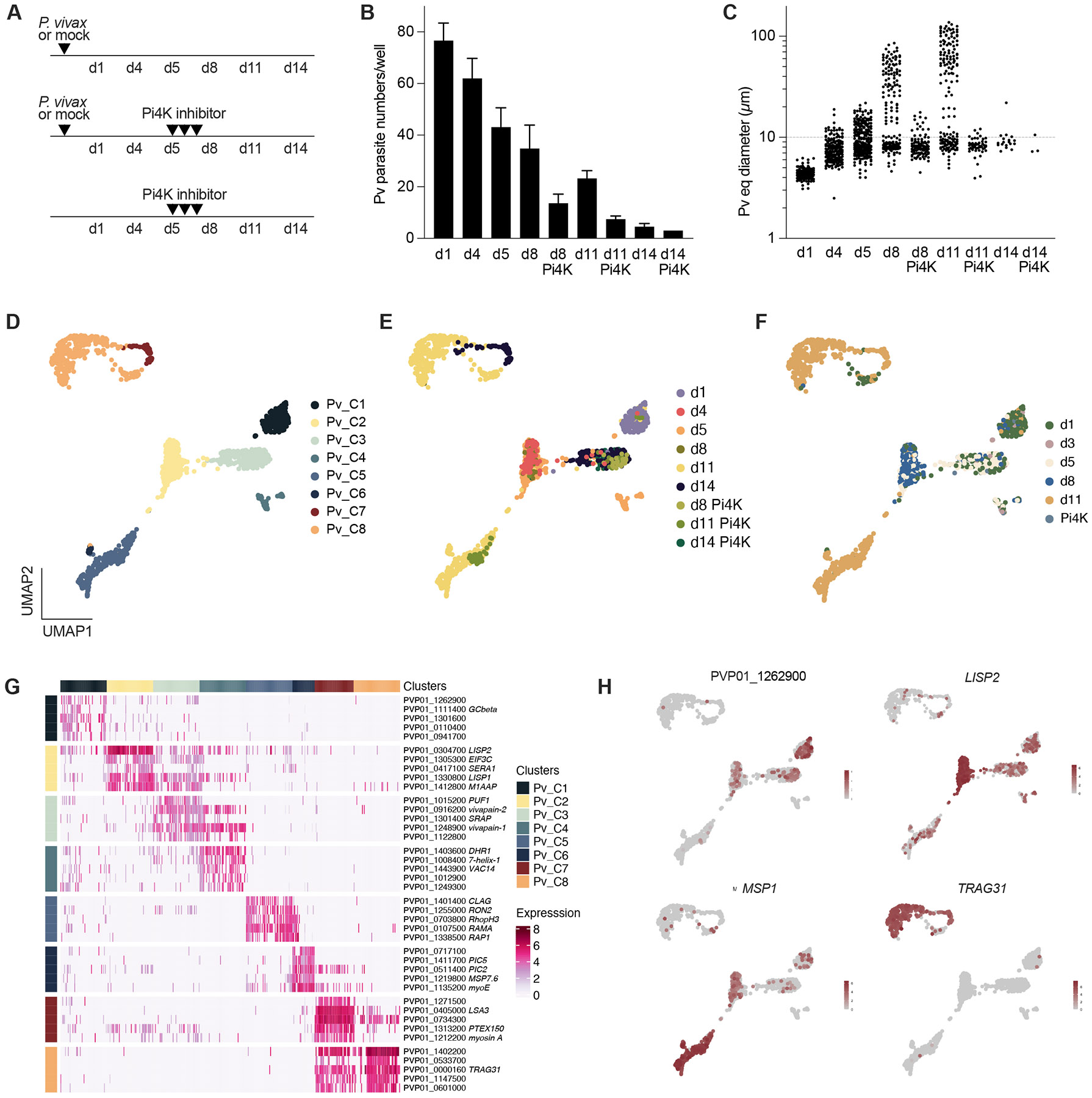
Single-cell profiling of *P. vivax* liver-stage infection. **A.** Timeline of infection, drug treatment and sample collection for scRNA-seq (d, day). PiK4 inhibitor treatment (d5-8) depletes replicative stages. **B-C.** Parasite numbers (**B**) and size distribution (**C**). Each dot represents an individual parasite (n = 3-8 wells pooled from 2 independent infections, n = 1 well shown for d14 Pi4K). The traced line indicates the 10 μm cut-off used for hypnozoite staging. The percentage of hypnozoites is as follows: d5, 62; d8, 46; d8 Pi4K, 83; d11, 36; d11 Pi4K, 90; d14, 78; d14 Pi4K, 67. **D-E.** UMAP of 1,494 individual *P. vivax* parasites coloured by parasite cluster type (**D**) and sample identity (**E**) using Seurat. Each dot represents an individual parasite. **F.** Predicted developmental stage based on Spearman correlations with samples collected in bulk and analysed by RNA-seq. **G.** Heatmap showing top 5 marker genes for each cluster. **H.** UMAP with expression of PVP01_1262900 conversed unknown function, *LISP2*, *MSP1* and *TRAG31* highlighted. See also Figure S1 and Tables S1, S2, S4.

To comprehensively profile *P. vivax* liver-stage infection, we collected cells from MPCC cultures of primary human hepatocytes at multiple time points following infection (Figures 1A-C). Sampling spanned the full liver-stage developmental period (days 1 to 11), comprising a mix of both replicating schizonts and non-replicative hypnozoites. To obtain hypnozoite-enriched samples, MPCCs were treated from days 5 to 8 with a phosphatidylinositol 4-kinase (Pi4K) inhibitor compound, a dosing regimen that eliminates the replicative parasites while preserving the dormant parasites (Gural et al., 2018). For hypnozoite-enrichment without drug treatment, we collected cultures on day 14. To obtain a baseline reading of the host transcriptome, naïve and mock-exposed MPCCs were prepared and collected in parallel. Mock samples were obtained by exposing MPCC cultures to non-infected mosquito material. Samples from two independent infections performed with patient-derived *P. vivax* sporozoites were processed for high throughput single-cell RNA sequencing (scRNA-seq) using Seq-Well S^3^ (Hughes et al., 2020). Samples were also collected in bulk for RNA sequencing (RNA-seq) analysis.

Because whole transcriptome scRNA-seq resulted in poor representation of *P. vivax* genes and transcripts, we incorporated an additional step whereby barcoded parasite transcripts were enriched using previously validated nucleic acid baits targeting the entire *P. vivax* genome (Gural et al., 2018). Capture and re-sequencing of samples remarkably increased the efficiency of *P. vivax* gene and transcript detection (Figure S1A). After quality control filtering (Figures S1B-C; Tables S1-2), we obtained single-cell transcriptomes from 1,494 individual parasites. The daily tally fairly reflects the number of hepatic infections, except for day 11 samples from which a higher number of parasites was recovered (likely representing free merozoites released from mature schizonts during sample processing).

### Single-cell profiling *P. vivax* liver infection defines stage-specific gene signatures

Clustering of parasite transcriptomes with Seurat yielded 8 *P. vivax* clusters, corresponding to distinct developmental liver-stages that were visualized by uniform manifold approximation and projection (UMAP; Figure 1D; Table S2). Early-stage parasites from day 1 collection assembled in a single cluster Pv_C1, while parasites collected on days 4 to 8 spread into separate clusters Pv_C2-4. Notably, Pv_C3-4 clusters comprise most of the Pi4K-enriched samples as well as day 14 cultures, and likely represent the hypnozoite parasite population. Late-stage liver schizonts (day 11) scattered across clusters Pv_C5-C8 (Figure 1E). The different samples and developmental stages are concordant with bulk RNA-seq profiling (Figures 1F, S1D; Table S3) and the rodent single-cell malaria atlas, respectively (Figure S1E).

Based on the gene patterns that define each cluster, early liver-stage parasites (Pv_C1) are largely characterized by the expression of conserved genes with unknown function as well as likely residual expression of sporozoite-specific genes (GO terms related gliding activity and cytoskeleton organization process) (Figures 1G-H; Table S4). The Pv_C2 cluster appears to represent a core mid-stage liver program, comprising well-characterized genes such as those encoding liver-specific protein 1 (*LISP1*) and 2 (*LISP2*) and genes implicated in housekeeping functions important for parasite growth such as translation (*EIF3C*) and metabolism (*ENO*, *LDH*). From Pv_C2 cluster, two branches appear to emerge, one comprising Pv_C3-4 and the other Pv_C5-6 clusters. *PUF1,* a Pumilio RNA binding protein known to be involved in translational repression, and genes encoding proteins with peptidase activity (*vivapain-1* and *-2*) appear in the top 5 marker genes for clusters Pv_C3-4 (Figure 1G). The Pv_C5-C6 clusters, on the other hand, are represented by GO terms related to mitotic division and adhesion of symbiont to host. Gene cluster identifiers in late-stage Pv_C5-C6 include several members of merozoite surface proteins (*MSP*), serine repeat antigen (*SERA*), rhoptry associated (*RAMA*) and rhoptry neck (*RON*) multigene families that are necessary for red blood cell invasion (Figure 1G). Finally, among late-stage parasites, Pv_C7-C8 represent a previously unidentified population of hepatic parasites that co-express merozoite- and gametocyte-specific genes. Marker genes for these clusters include multiple copies of the tryptophan-rich protein family (which are expressed by merozoites of multiple *Plasmodium* species and have been implicated in erythrocyte invasion) the gametocyte antigen *G27/25,* the gamete release protein (*GAMER*) and the homolog of gametocyte exported protein 5 (*GEXP5*, aka *PHISTc*). Interestingly, several members of the apicomplexan AP2 family of transcription factors (AP2 TFs) were found to be highly expressed all throughout the liver-stage developmental phase. Multiple members appear specifically assigned to individual clusters, suggesting distinct roles that could contribute to the observed cascade of differential gene expression in the liver (Figures 1G, S1F).

### Transcriptional profiling of *P. vivax* liver-stages reveals early sexual commitment

**Figure 2.**
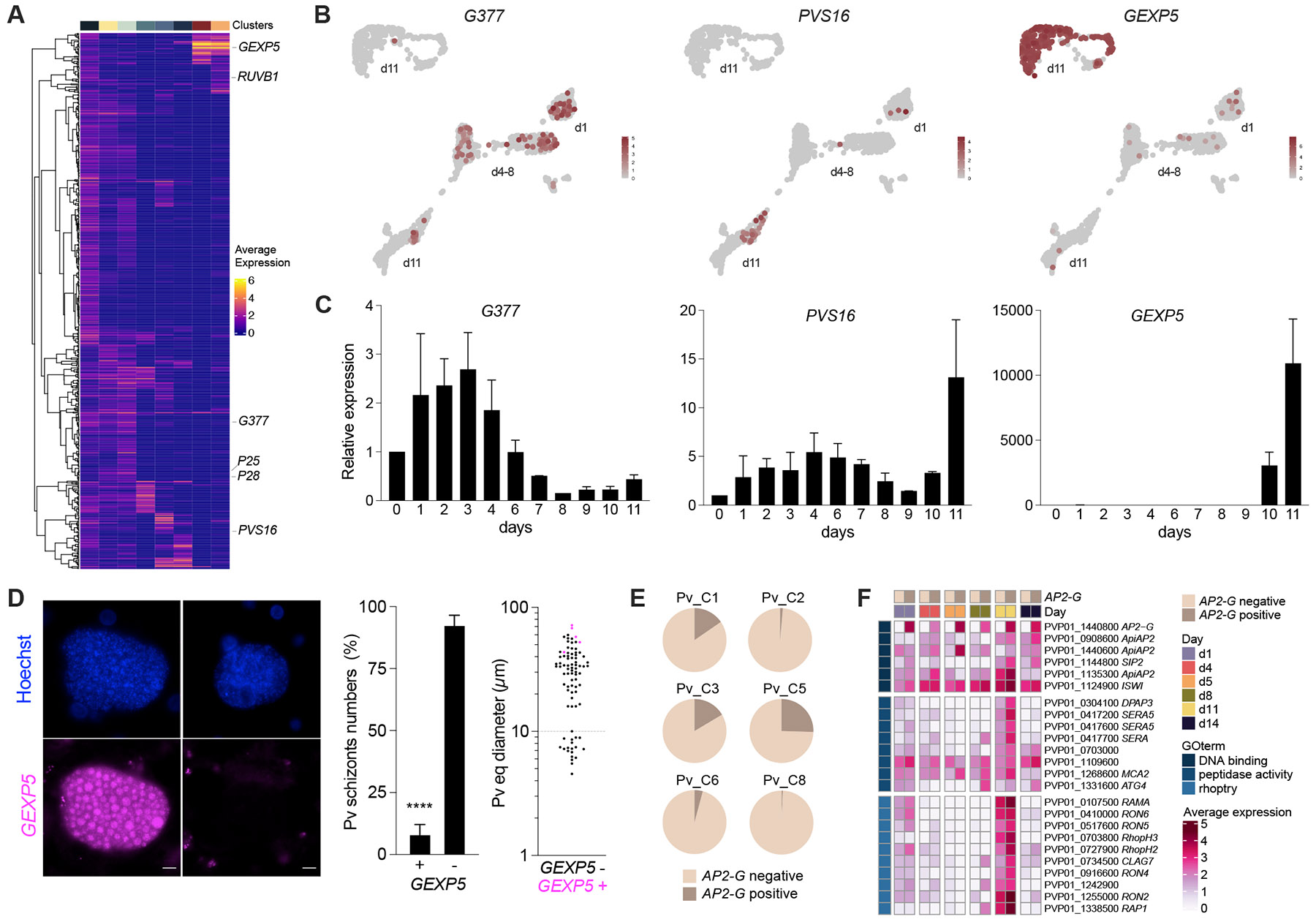
Characterization of *P. vivax* signature of hepatic sexual commitment. **A.** Average expression within each cell cluster of *P. vivax* orthologs of 591 *P. falciparum* genes previously associated with sexual development, from sexual ring stages to mature gametocytes (Pelle et al., 2015). Parasite clusters indicated on top are temporarily ordered as in Figure 1G. **B-C.** UMAP and relative expression of *G377*, *PVS16* and *GEXP5* by quantitative RT-PCR (mean ± SEM; n = 2 independent infections). The 3 genes have been shown to be direct AP2G targets. **D.** RNA *in situ* hybridization of *P. vivax* parasites at day 10. GEXP5 transcripts shown in magenta. Scale bars, 10 μm. Parasite numbers and size distribution of *GEXP5*-positive and -negative schizonts (mean ± SEM; n = 4 wells; t-test: ****, p < 0.0001). **E.** Pie charts showing the proportion of *AP2-G* positive parasites by cluster. The values are as follows: Pv_C1, 15.4%; Pv_C2, 1.4%; Pv_C3, 16.5%; Pv_C5, 25.5%; Pv_C6, 4.2%; Pv_C8, 0.7%; Pv_C4 and Pv_C7, 0%. **F.** Average expression of differentially expressed genes between *AP2-G* positive and *AP2-G* negative parasites by infection time point. The genes shown are associated with the top GO terms.

To gain further insight into the subpopulation of parasites defined by clusters Pv_C7-C8, we searched the dataset for additional gametocyte-specific genes obtained from various blood-stage datasets (Obaldia et al., 2018; Pelle et al., 2015; Sà et al., 2020). Expression of canonical sexual markers (*PVS16, P28, P25),* including female-specific (*RUBV1* and *G377*), mature and immature gametocyte-specific genes were detected throughout the developmental liver-stages in multiple clusters (Figure 2A). Differential expression patterns were confirmed by quantitative RT-PCR analysis for a small subset of gametocyte genes (Figures 2B-C). For further validation, we performed *in situ* hybridization for *GEXP5*, which appears highly expressed in day 11 samples (Figures 2B-C) and is known as an early sexual stage marker in blood-stages of the human malaria parasite *P. falciparum* (Tibúrcio et al., 2015). We found *GEXP5* transcripts in 5-15% of schizonts, with positive signal detected in all individual nuclei of the segmented schizont (Figure 2D).

Moreover, we found that up to 25% of parasites across different clusters express the gene encoding AP2-G, the transcriptional master regulator of sexual development in *Plasmodium* blood stages (Kafsack et al., 2014; Sinha et al., 2014) (Figure 2E). Exploring the dynamics of this transcription factor, we found that the proportion of cells expressing *AP2-G* peaked on days 1, 5, 11 and 14 (Figure 2F), a wave-like pattern reminiscent of that observed in blood-stage parasites undergoing sexual commitment (Poran et al., 2017). A set of genes encoding other AP2 TFs, and the chromatin remodeler (ISWI) appear to follow a similar pattern of transcription, suggesting that AP2-G might coordinate with other transcriptional regulators to accomplish sexual commitment in the liver (Figure 2F). On day 11, AP2-G induction is also linked to genes implicated in egress and invasion, as seen previously for blood-stages (Josling et al., 2020).

Our findings corroborate that commitment to gametocytogenesis might occur early during liver-stage development in a subset of parasites, likely leading to formation of sexually committed merozoites. These results are consistent with previous studies that detected PVS16 protein in a fraction of *P. vivax* hepatic schizonts (Roth et al., 2018; Schafer et al., 2020), and others that found gametocyte-specific transcripts in the blood of volunteers infected with *P. vivax* by mosquito bites (Adapa et al., 2019). In the controlled human infection, gametocyte transcripts were identified in the top-ranking genes as early as day 9 after infection, when the first merozoites emerge from the liver.

### Non-replicative *P. vivax* liver-stages depend on proteolytic activity and are sexually committed

**Figure 3.**
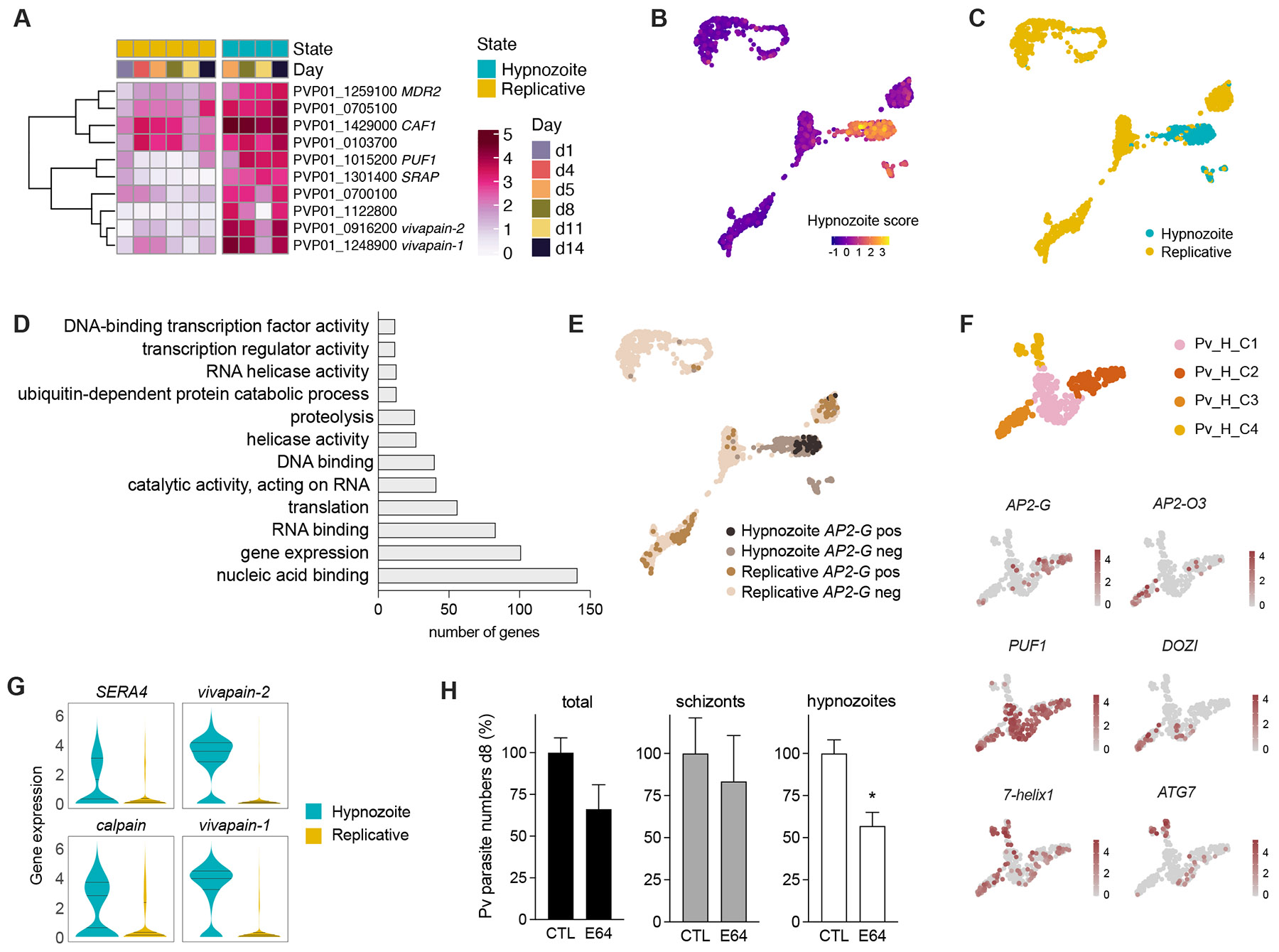
Characterization of *P. vivax* hypnozoite-stage transcriptomes. **A.** Average expression of the 10 genes in the hypnozoite score. **B-C.** UMAP with cells coloured by their hypnozoite score (**B**). Cells with a score > 0.75 were labelled as hypnozoite (**C**). **D.** GO term analysis of genes expressed in hypnozoite versus replicative. **E.** UMAP with hypnozoite/replicative states and *AP2-G* expression highlighted. **F.** UMAP of hypnozoite clusters (re-clustering Pv_C3-C4). Gene markers for each hypnozoite subcluster shown on bottom. **G.** Violin plot showing the expression of 4 cysteine peptidases upregulated in hypnozoite compared to replicative parasites. **H.** Treatment (d0-8) of *P. vivax* infected cultures with protease inhibitor E64 (1 μM; mean ± SEM; n = 3-5 wells; t-test: *, p < 0.05). See also Figure S2.

To inform the identification of hypnozoite-specific gene signature(s), we first compared the distribution of non-treated versus the Pi4K-treated hypnozoite-containing samples across the UMAP, which revealed that the only cluster significantly enriched for Pi4K-treated cells was Pv_C3. This enabled us to calculate a hypnozoite score based on the top markers of that cluster and precisely define replicative and non-replicative hypnozoite states across the UMAP (Figures 3A-C, S2A). Hypnozoites were significantly enriched in genes encoding proteins with proteolytic activity (26 genes) and nucleic acid binding activity (141 genes), including TFs, DNA and RNA binding, translation, and helicase activity (Figure 3D; Table S4). Remarkably, the multidrug resistant MDR2 gene appears in the top scoring genes, which could explain the insensitivity of hypnozoite stages to most antimalarial treatments.

Among the TFs, we found expression of gametocytogenesis regulators *AP2-G*, *AP2-FG* (Yuda, 2020), and *AP2-O3* (Li et al., 2021) suggesting that a subpopulation of *P. vivax* hypnozoites could be pre-committed to become sexually transmissive forms. Presence of gametocyte-specific transcripts in hypnozoite-enriched samples was confirmed by quantitative RT-PCR analysis (Figure S2B). By combining the hypnozoite score with *AP2-G* expression as proxy for sexual commitment, we further identified distinct parasite populations and states: i) replicative *AP2-G* positive, ii) hypnozoite *AP2-G* positive, iii) replicative *AP2-G* negative, and iv) hypnozoite *AP2-G* negative, which exhibit varying proportions during liver-stage development (Figure 3E).

To further explore transcriptional differences among individual hypnozoites, we next re-clustered Pv_C3-4, which revealed four diverse subclusters with a distinctive pattern of AP2 TFs expression (Figures 3F; S2C). Interestingly, the Pv_H_C4 sub-cluster appears to be depleted of markers of translational repression *PUF1* and *DOZI*, but significantly enriched in a gene encoding a 7-helix protein. This protein has been postulated to operate in the re-initiation of translation of repressed transcripts during *P. falciparum* gametogenesis (Bennink et al., 2018). Other marker genes for this subcluster are *ATG7*, which encodes a protein necessary for parasite growth in *P. falciparum* blood-stages (Walker et al., 2013), and *PHD1*, a member of the GCN5 histone acetyltransferase complex that controls expression of erythrocyte invasion genes (Miao et al., 2021). The Pv_H_C4 sub-cluster could therefore represent a subpopulation of *P. vivax* hypnozoites undergoing reactivation, i.e. shifting from dormancy to schizogony.

Protein degradation is important for maintenance of a quiescent state induced by nutrient limitation during the *P. falciparum* erythrocytic cycle (Babbitt et al., 2012). To functionally validate our findings and determine whether proteolytic activity (Figure 3G) could be required to maintain viability of quiescent hypnozoites, we exposed *P. vivax*-infected MPCCs to E64, a membrane-permeable protease inhibitor. No effect was found on schizont forms; however, the number of hypnozoites was significantly reduced in the E64-treated cultures (Figures 3H, S2D). Altogether these results suggest that *P. vivax* hypnozoites in the liver could represent a reservoir of sexually committed parasites and dormancy might depend on transcription and translational repression mechanisms and proteolysis for long-lasting viability.

### Dormancy and hepatic sexual differentiation are associated with distinct host metabolic states

**Figure 4.**
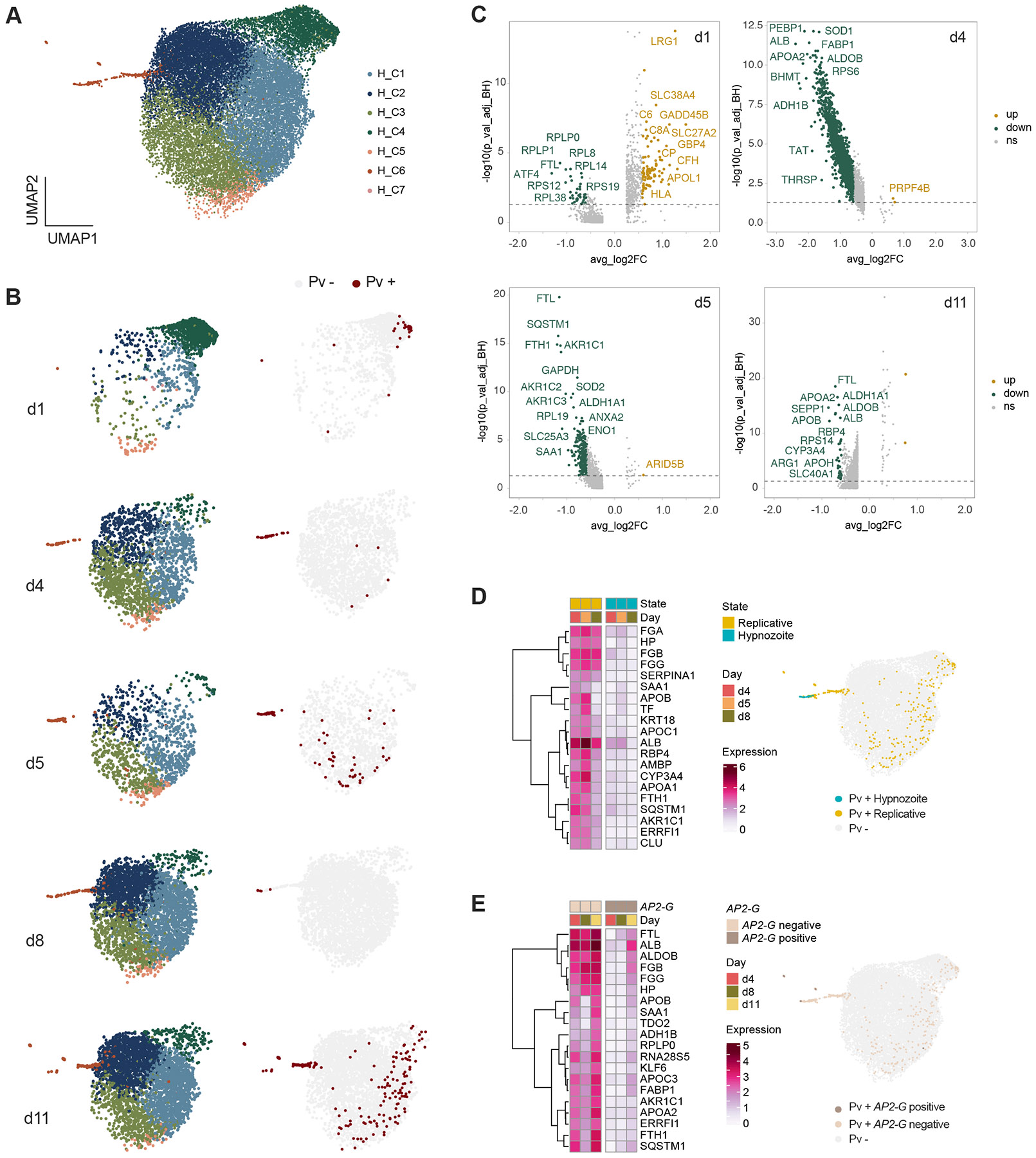
Dual scRNA-seq analysis of *P. vivax* liver-stage infection. **A.** UMAP of 33,379 individual cells coloured by cell type cluster using Seurat. **B.** UMAP of 12,841 *P. vivax*-exposed hepatocytes showing distribution of the different samples (left) and infection status (right). *P. vivax*-positive hepatocytes coloured in dark red. **C.** Volcano plots showing differentially expressed genes in *P. vivax*-positive and -negative cells. Genes coloured in green or yellow show a fold change of at least 1.5 (FDR < 0.05). No significantly different genes detected on day 8. **D-E.** Average expression of the top 20 differentially expressed genes (FC > 4 and FDR < 0.05) between infected host cells carrying replicative versus non-replicative parasites (**D**) and *AP2-G* positive versus *AP2-G* negative parasites (**E**) shown at the indicated infection time points. UMAPs of the different cell types shown on the right. See also Figure S3 and Table S5.

To characterize host cell signatures linked to infection and host-dependent differences in parasite transcriptomes, we profiled samples of unexposed, mock- and *P. vivax*-exposed MPCCs across all timepoints (Figure 1A). From the 33,379 high-quality human transcriptomes obtained after quality control filtering (Figure S3A; Table S2), we subset the *P. vivax*-exposed samples across all timepoints, corresponding to 12,841 cells. Clustering using Seurat revealed 7 human clusters (H_C1-7) with differential enrichment in *P. vivax-*associated hepatocytes (Figures 4A-B). Cluster H_C2 harbors only a small number of infected hepatocytes, while H_C6 appears highly enriched in infected cells.

Comparing *P. vivax*-positive and -negative cells demonstrated that infected cells are transcriptionally distinct, in a time-dependent manner (Figure 4C; Table S5). GO term enrichment analysis revealed strong activation of innate immune defense genes during early infection and suppression of liver-specific functions in *P. vivax* infected cells in later infection. Examples of differentially expressed genes on day 1 include markers of inflammation and stress response, such as *LRG1*, *TNFSF10*, *NFKB1,* and the family of acute-phase serum amyloid A proteins (*SAA-1, -2, -4*) with increased expression in the *P. vivax-*infected cells. LRG1 is a recently identified biomarker able to distinguish between malaria and dengue infection (Kumar et al., 2020). A significant number of transporters, the complement and lipid metabolic processes appear induced in the infected population at this timepoint. Notably, the cholesterol transporter *SRB1* was found expressed in higher levels in 63% of the infected cells on day 1 (Figure S3B), consistent with a recent report identifying it as host factor for *P. vivax* parasites (Manzoni et al., 2017). Other host factors previously identified in other species do not show significant differential expression (Figure S3B; Table S5). Later in infection, however, interferon (IFN) response and lipid metabolic processes become suppressed. Other significant GO terms appearing dysregulated include apoptosis, translation, and viral replication. These results are consistent with a model suggesting shut down of hepatic function during infection. Albumin (*ALB*), arginase 1 (*ARG1*) and fatty acid binding protein 1 (*FABP1*) appearing in the top 10 of most downregulated genes. IFN-related genes with significant low expression in later infection, such as *ISG15* and *IFI6,* might be involved in creating a permissive environment for late-stage parasite development and replication.

Given that sexual differentiation and gametocytogenesis in malaria parasites have been linked to host-derived physiological signals (Brancucci et al., 2017), we next compared the transcriptomes of infected hepatocytes based on the sexual and replicative status. To identify hepatocytes hosting replicative and non-replicative parasites, we applied the hypnozoite score defined in Figure 3. For the sexual state, we compared *AP2-G*-positive and *AP2-G*-negative *P. vivax* parasites. Interestingly, we found that hepatocytes bearing non-replicative or *AP2-G* positive parasites exhibit significant lower expression of genes encoding proteins implicated in iron transport and storage (*TF, FTL, FTH1*) and lipid metabolism (*APOA1, APOC1, APOB, FABP1, CYP3A4*). Genes encoding albumin (*ALB*), haptoglobin (*HP*) and alpha-1-antitrypsin (*SERPINA1*) proteins are also among the top 20 of differentially expressed (Figures 4D-E; Table S5). These observations point to common mechanisms, suggesting that deficiency in specific intracellular metabolites (possibly iron or lipids) could serve as triggers of both quiescence and sexual commitment.

### Interferon responses in uninfected bystander hepatocytes

**Figure 5.**
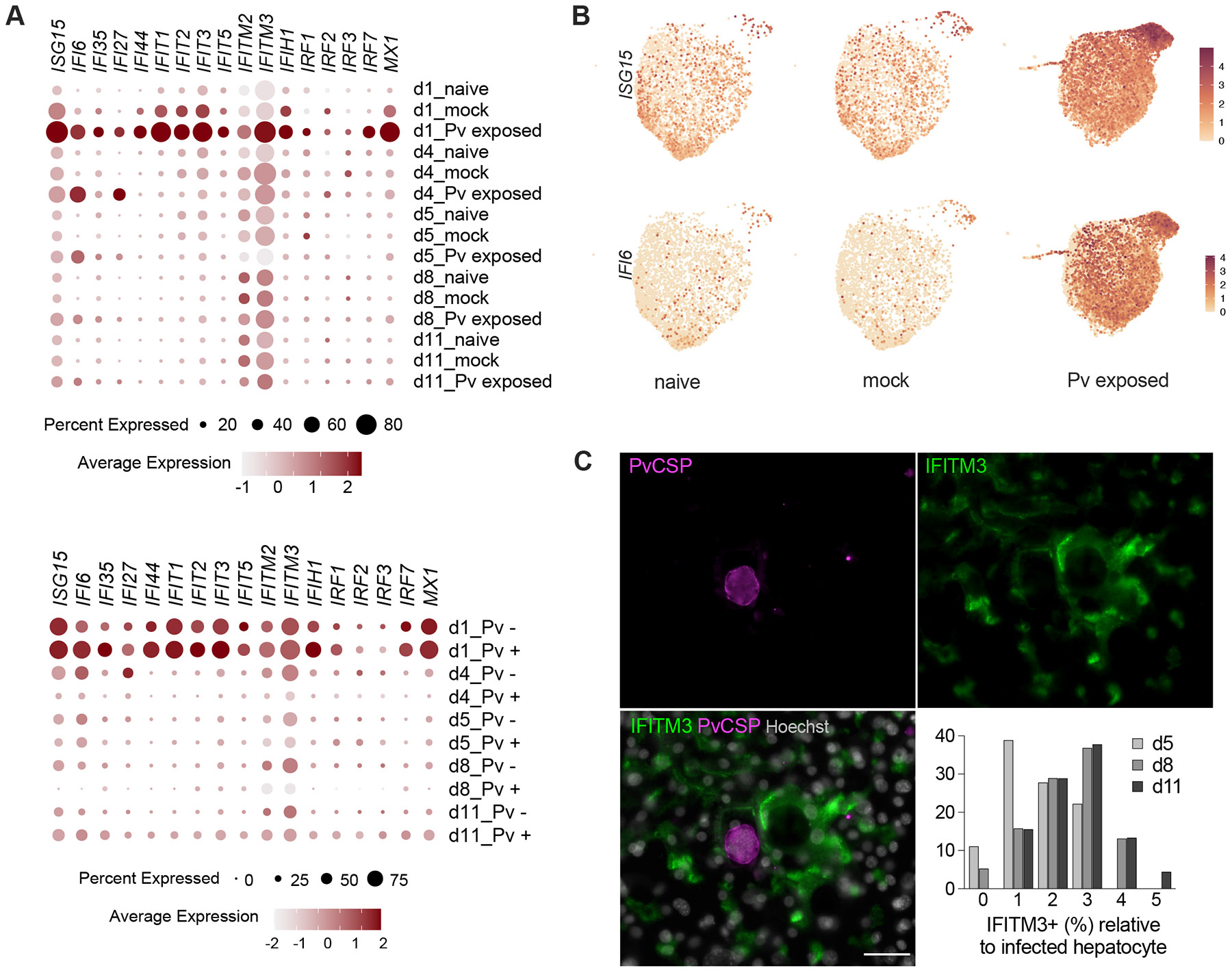
IFN responses in *P. vivax*-infected and bystander hepatocytes. **A.** Dot plots showing expression of IFN-related genes throughout infection time course in naïve, mock-exposed and *P. vivax*-exposed samples (top) and in the *P. vivax*-exposed samples by infection status (bottom). **B.** UMAP highlighting *ISG5* and *IFI6* expression levels. **C.** Expression of IFITM3 protein (green) in *P. vivax*-infected cultures. Representative images of parasites on day 8. Scale bars, 50 μm. Bar plot shows frequency distribution of IFITM3-positive hepatocyte layer relative to the infected cell by infection timepoint. See also Figure S4.

Taking advantage of our single-cell approach, we also investigated the impact of *P. vivax* infection in the bystander hepatocytes. Comparing the transcriptomes of cells exposed to *P. vivax* to mock-exposed or naïve hepatocytes, we detected a widespread induction of the alpha/beta IFN response, mainly in the *P. vivax-*exposed uninfected cells (Figures 5A-B; S4). Day 1 samples stand out given that the induction of multiple IFN-responsive genes and gene families, including *IRF7*, *IFI6*, *IFI27, IFIT1-5, IFI44* and *IFIH1*, is observed in more than 40% of the hepatocytes. Transcripts of effector IFN-induced cell membrane proteins *IFITM2* and *IFITM3* are increased throughout the infection time course (Figures 5A-B). While the strong induction observed at early time points could be linked to the sporozoite cell traversal and wounding of cells prior infection (Mota et al., 2001), the temporal order and gene composition of the IFN response to *P. vivax* is remarkably similar to that of hepatitis C virus (HCV) infection (Sheahan et al., 2014), suggesting rather a common hepatocyte defense mechanism in response to human hepatotropic pathogens.

To validate our findings with an independent approach, we performed immunofluorescence analysis of IFITM3 in *P. vivax*-infected MPCCs. We found that IFITM3 protein localizes in the bile canaliculi and tight junctions and confirmed the upregulation of IFITM3 in the uninfected bystander cells, with higher expression detected in the vicinity of the infected hepatocytes (Figure 5C). IFITMs restrict infections by many viruses, employing diverse mechanisms (Diamond and Farzan, 2013). Based on its localization in the host membrane and lack of association with the parasite membrane, IFITM3 is likely to be involved in gap junction communication and propagation of host responses from infected to uninfected cells (Luther et al., 2020; Patel et al., 2009).

### Host interferon responses control *P. vivax* infection

**Figure 6.**
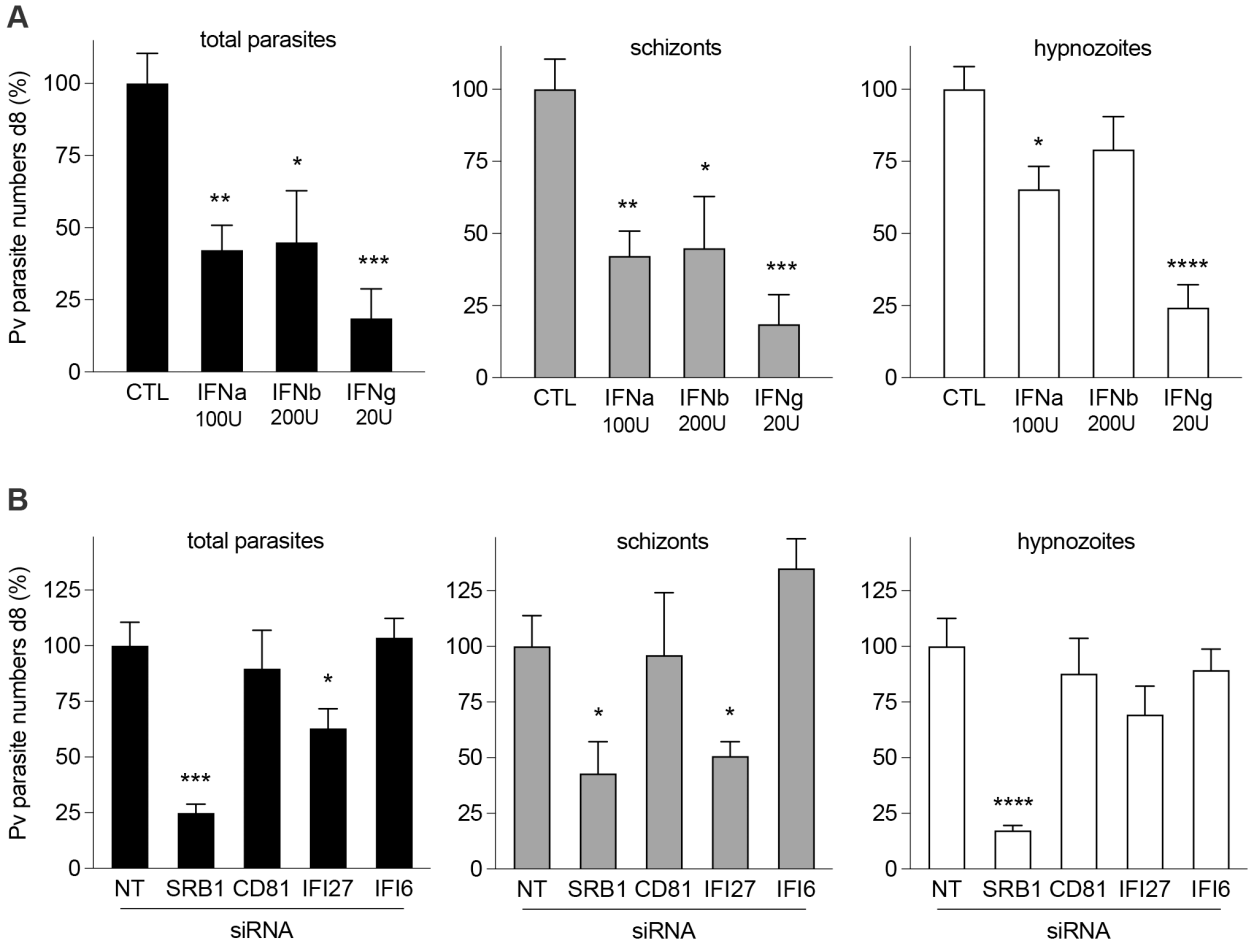
IFN responses control *P. vivax* hepatic infection. **A.** IFN treatment (d5-8) of *P. vivax*-infected MPCC cultures. **B.** siRNA treatment prior infection (d -3). SRB1 and CD81 were known host factors for *P. vivax* and *P. falciparum* and were used as controls. Bar plots show quantification of parasite numbers at day 8 (mean ± SEM; n = 5-6 wells pooled from 2 independent infections; 1-way ANOVA test: *, p < 0.05; **, p < 0.01; ***, p < 0.001; ****, p < 0.0001). See also Figure S5.

Given the innate immune responses observed during early *P. vivax* infection (Figures 4-5), we hypothesized that the activation of this pathway could be associated with the significant drop in parasite numbers observed from day 1 to 11 (Figure 1B). To assess the impact of activated IFN signaling and provide mechanistic insight into IFN-mediated responses on *P. vivax* infection, we employed two approaches: i) treatment with IFN cytokines and ii) silencing of downstream effector proteins. For the first approach, *P. vivax*-infected MPCCs were treated with IFN alpha (IFNa) and beta (IFNb) cytokines. As control, we used IFN gamma (IFNg), which is known to reduce *P. vivax* infection by activating autophagy (Boonhok et al., 2016; Ferreira et al., 1986). While no effect was detected in terms of the kinetics of parasite development (Figure S5A), the number of both *P. vivax* schizonts and hypnozoites were significantly reduced upon treatment with IFNa. IFNb appeared to have a stronger effect on schizont stages, by contrast IFNg treatment revealed a potent anti-hypnozoite activity (Figure 6A).

For the gene silencing approach, we utilized siRNA oligonucleotides to specifically reduce expression of IFI6 and IFI27 prior to infection. IFI6 and IFI27 belong to the same family of IFNa-induced effector proteins exerting opposing activities on apoptosis. They can exhibit antiviral activity depending on the virus (Qi et al., 2015; Ullah et al., 2021). Both *IFI6* and *IFI27* transcripts were increased upon infection with *P. vivax,* however only 23% of the *P. vivax* positive cells co-expressed the 2 genes (Figures 5A, S5B-C). Consistent with an antagonistic function, the proteins they encode for appear to have opposing effects on infection. Reduction of IFI6 in MPCCs led to a modest increase in the numbers of *P. vivax* schizonts, while reduction of IFI27 showed a significant decrease in the numbers of schizonts. No effect was found on parasite size or hypnozoite numbers (Figures 6B, S5A). These results support an antiparasitic role for IFI6, likely promoting parasite elimination by activation of apoptosis in the host cell. IFI27 on the other hand appears to enhance *P. vivax* infection in human hepatocytes. Our siRNA experiment also validated the postulated role of SRB1 as a species-specific entry host factor for *P. vivax* (Manzoni et al., 2017) (Figure 6B). Collectively, our results suggest that successful *P. vivax* infection likely involves robust mechanisms for subverting host IFNa/b responses, which are distinct in the hepatocytes harboring dormant hypnozoites.

## DISCUSSION

Here we comprehensively surveyed the molecular composition of *P. vivax* parasites in distinct liver-stages, as well as host or bystander hepatocytes, throughout the course of infection. The presented data enabled us to assemble a single-cell liver atlas of relapsing human malaria. Leveraging the portability of two established platforms (MPCC and Seq-Well) to culture and collect individual human hepatocytes infected in an endemic location, we performed dual single-cell sequencing analysis and further developed a method to selectively enhance capture of parasite transcripts. We demonstrated the robustness and utility of this approach by sequencing 1,494 *P. vivax* parasites and 33,383 hepatocyte transcriptomes and provided validations and mechanistic insights for the identified gene signatures and parasite-host interactions.

Dual transcriptional profiling of *P. vivax* infection revealed host- and stage-dependent gene expression patterns in both parasites and hepatocytes. Our data is consistent with the following model: upon invasion, a subset of *P. vivax* sporozoites in cells with reduced hepatic metabolism activate *AP2-G*. Of those, a fraction will remain dormant, while the remaining portion will activate the schizogony program. Thus, at the point of egress, two populations of hepatic *P. vivax* merozoites will emerge from the liver: an asexual population developing into blood-stages, and a sexually committed population developing into gametocytes for mosquito transmission (Figure S6). This model, while distinct from the existing understanding of the *Plasmodium* life cycle, is in agreement with historical observations of rapid *P. vivax* transmission that occur before the onset of symptoms (Baker, 2010), and is consistent with the high abundance of gametocyte transcripts detected in blood collected from malaria patients (Adapa et al., 2019; Kim et al., 2019). In our model, parasites that do not activate schizogony, hypnozoites, remain in a dormant state via transcriptional/translational repression mechanisms and rely on proteolytic activity to sustain viability. Bypassing the need for an asexual replication phase might represent an evolutionary imperative to preserve genome integrity, given the prolonged period between infection and transmission. From a clinical perspective, our results advocate for the use of gametocyte-targeting molecules as a way to reduce the hypnozoite reservoir in the liver. Supporting this strategy, a recent *in vitro* drug screen reported anti-hypnozoite activity for several previously identified *P. falciparum* gametocidal compounds (Maher et al., 2021). An alternative would be the development of a “commit and kill” clinical approach. Instead of employing epigenetic modulators to first awake the dormant parasites and then kill the replicating forms with a conventional drug as previously proposed (Dembélé et al., 2014), we would leverage drugs that enhance gametocyte commitment and then use a gametocidal drug to kill. Parasite-specific protease inhibitors could also be screened and investigated for anti-relapsing activity.

Additionally, our work reveals dysregulation of IFN and inflammatory signaling pathways in *P. vivax*-infected hepatocytes. After a strong initial activation, the reduced expression of IFN-responsive genes in infected cells suggests a protection mechanism for the parasite from host cell-mediated killing. Downregulation of IFN signaling may also contribute to under-activation of adaptative immune responses by antigen presenting cells, leading to parasite persistence in the organism. The mechanisms by which IFN suppression is achieved in infected cells or the impact of host genetics and host-dependent IFN responses, which are key determinants of clinical outcome in HCV infections (Sheahan et al., 2014), remain unknown. Nevertheless, induction treatments with IFN and gene silencing experiments in our immune-cell free microliver system began unveiling restricting and enhancing factors of *P. vivax* infection. Furthermore, we described upregulation of innate immune response pathways in uninfected bystander cells, similar to those observed in viral infections that help control spreading of the virus to neighboring cells (Kotliar et al., 2020; Sheahan et al., 2014). In the context of malaria, this phenomenon might prime or protect cells from a secondary infection, as suggested by the inhibition of malaria re-infection by IFN in a rodent model of *Plasmodium* (Liehl et al., 2013, 2015). Future studies employing spatial single-cell transcriptomics could reveal *P. vivax*-specific gene signatures and spatially heterogeneous responses in bystander cells.

In summary, this study presents a transcriptional description of individual patient-derived *P. vivax* liver-stages, their host cells and uninfected bystander cells over the course of infection in human hepatocytes. Leveraging multiple single-cell technologies (host-pathogen sequencing, *in situ* hybridization, and immunofluorescence), we reveal earlier than expected sexual commitment during *P. vivax* liver-stage development in both replicative and non-replicative parasites, and a dominant innate immune response that exhibits distinct signatures in infected and uninfected bystander hepatocytes. Taken together, these clinically relevant insights provide a framework for characterizing host-parasite interactions in *P. vivax* infections. We expect the methods described here to be applicable for profiling not only other *Plasmodium* parasites at various stages of the life cycle, but also other intracellular pathogens where low abundance of transcripts or host contamination make it difficult to perform single-cell studies.

## STAR METHODS

### RESOURCE AVAILABILITY

#### Lead contact

Further information and requests for resources and reagents should be directed to and will be fulfilled by the lead contact, Sangeeta N. Bhatia (sbhatia@mit.edu).

#### Materials availability

Antibodies generated in this study will be shared by the lead contact upon request.

#### Data and code availability

Bulk RNA-seq and scRNA-seq data have been deposited at GEO (GSE197459). Gene-by-cell expression matrices have been deposited at Zenodo (doi:10.5281/zenodo.6280956) and will be publicly available as of the date of publication. Any additional information required to reanalyze the data reported in this paper will be available from the lead contact upon request. Microscopy data reported in this paper will be shared by the lead contact upon request.

### EXPERIMENTAL MODEL AND SUBJECT DETAILS

#### Fibroblasts and primary cell cultures

J2-3T3 male murine embryonic fibroblasts (gift of Howard Green, Harvard Medical School) were cultured at < 20 passages in medium comprising of Dulbecco’s Modified Eagle Medium (DMEM, Corning), 10% (v/v) bovine serum (Thermo Fisher Scientific), and 100 mg/mL penicillin-streptomycin (Corning) and were kept at 37°C in a 5% CO2 environment.

Cryopreserved primary human hepatocytes were purchased from BioIVT, a vendor permitted to sell products derived from human organs procured in the United States of America by federally designated Organ Procurement Organizations. Human hepatocytes (male donor, age 57) were maintained in DMEM with 10% fetal bovine serum (FBS, GIBCO), 1% ITS (insulin/transferrin/selenous acid and linoleic acid, BD Biosciences), 7 ng/mL glucagon (Sigma-Aldrich), 40 ng/mL dexamethasone (Sigma-Aldrich), 15 mM HEPES (GIBCO), and 100 mg/mL penicillin-streptomycin (Corning). Hepatocyte cultures were kept at 37°C in a 5% CO2 environment.

#### *P. vivax* parasites

*Anopheles dirus* mosquitoes were fed on blood collected from symptomatic patients attending malaria clinics in Tak, Songkla, and Ubon-Ratchathani Provinces in Thailand, confirmed positive for only *P. vivax* via microscopy and RT-PCR. The *P. vivax* infected blood samples were collected from patients under the approved protocol by the Ethics Committee of the Faculty of Tropical Medicine, Mahidol University (MUTM 2018-016). The written informed consent was obtained prior to blood collection.

Briefly, *P. vivax* infected blood was drawn into heparinized tubes and kept at 37°C until processing. Infected blood was washed once with RPMI 1640 incomplete medium. Packed infected blood was resuspended in warm non-heat inactivated naive human AB serum for a final hematocrit of 50%. Resuspended blood was fed to laboratory reared female *Anopheles dirus* mosquitoes for 30 minutes via an artificial membrane attached to a water-jacketed glass feeder kept at 37°C. Engorged mosquitoes were kept on 10% sugar at 26°C under 80% humidity at the designated insectary at the Mahidol Vivax Research Unit. Sporozoites were dissected from the salivary glands of infected mosquitoes 14-21 days after blood feeding and pooled in DMEM supplemented with 200 mg/mL penicillin-streptomycin.

### METHODS DETAILS

#### MPCCs and *P. vivax* infection

Primary human hepatocytes were seeded on collagen-micropatterned 96-well plates and surrounded with murine embryonic fibroblasts 3T3-J2s as detailed previously (March et al., 2015). For the scRNA-seq analysis, MPCCs were established using 3T3-J2s expressing an inducible apoptosis switch (Chen et al., 2020). MPCCs were infected with fresh sporozoites obtained through hand dissection of *P. vivax*-infected mosquitoes. For mock samples, MPCCs were exposed to material from non-infected mosquito salivary glands (matched number of dissected mosquitoes and processed similarly as the infected counterparts). Naïve samples received the culture media used for the infections.

#### siRNA and drug treatments

To obtain hypnozoite-enriched samples, *P. vivax*-infected MPCCs were dosed with a schizont-specific drug Pi4K inhibitor (MMV390048, 1 μM) for 3 days starting on day 5 after infection. Similar dosing schedule was used for treatments with IFN alpha 2a, beta and gamma. E64 treatment started at 4h post-infection after media washing the cultures.

siRNA oligonucleotides were added to patterned hepatocytes as described previously (Mancio-Silva et al., 2019). ON-TARGETplus SMARTpool siRNA oligonucleotides (Dharmacon) were delivered to hepatocytes by using RNAiMAX Transfection Reagent (Thermo Fisher Scientific) per manufacture’s protocols at final concentration 50 nM. *P. vivax* sporozoites were added at day 3 post siRNA treatment.

#### Seq-Well and *P. vivax* capture

For the scRNA-seq analysis, an updated Seq-Well protocol including a second-strand synthesis step was employed (Hughes et al., 2020). Briefly, MPCCs were first treated with B/B Homodimerizer (0.5 μM) for 30 minutes at 37°C to partially deplete the fibroblast cells via apoptosis (Chen et al., 2020). After washing, the cultures were dissociated by Trypsin (0.25%) 5-minute treatment at 37°C. A suspension with 10,000 - 15,000 cells (pooled from duplicate MPCC wells) was then loaded onto a functionalized-polydimethylsiloxane array preloaded with uniquely barcoded mRNA capture beads. For each time-point in the two independent infections, two separate arrays were loaded with *P. vivax*-infected samples, and single arrays for mock and naïve samples. After cells had settled into wells, the array was sealed with a hydroxylated polycarbonate membrane with a pore size of 10 nm, facilitating buffer exchange while permitting cell lysis, mRNA transcript hybridization to beads, and bead removal before proceeding with reverse transcription. The obtained bead-bound cDNA product then underwent Exonuclease I treatment to remove excess primer before proceeding with second-strand synthesis and PCR amplification. Illumina sequencing (NOVAseq) was performed for one of the two infection batches.

To capture parasite reads from Seq-Well, full length cDNAs were amplified with an additional 5 cycles using Kapa HiFi polymerase (3-minute extension time was used to increase concentration for capture). 200 - 300 ng of cDNA was concentrated using a speedvac, reconstituted in 3.4 μL water and hybridized for 32 hours as previously described (Gural et al., 2018). 10 μL of capture material was amplified for 15 cycles following the standard protocol. Captured cDNA was then prepared into Illumina libraries using NexteraXT (Illumina). Final libraries were quality controlled using Fragment Analyzer (Agilent) and qPCR prior to Illumina sequencing (NextSeq500). Sequencing of captured parasite transcripts was performed for the two infection batches.

#### Tri-genome mapping target generation

Chromosomes and contigs from human (hg19), murine (mm10) [same ENSEMBL releases as in (Macosko et al., 2015)] and *P. vivax* genome (PvP01 v1 release) were renamed with a species-specific prefix and concatenated into a fasta file. Corresponding gtf files were adapted to match prefixed chromosome names, and genes names were adjusted as an ENSEMBLID_SPECIES_GeneSymbol concatenated string.

#### scRNA-seq processing

For each sample, fastq files originating from multiple sequencing runs were concatenated and processed using an analytical pipeline derived from the DropSeq pipeline v. 1.12, as described in (Gierahn et al., 2017) (https://github.com/broadinstitute/Drop-seq). Briefly, reads were converted to a bam file using picard v. 2.9.0-1-gf5b9f50-SNAPSHOT, tagged with cell and transcript barcodes, and subsequently sequencing adapters and polyadenosine tracts were trimmed. Upon regenerating fastq files, reads were aligned with STAR v. 2.5.3a (Dobin et al., 2013) against the aforementioned tri-genome reference. Genomic features of the aligned reads were annotated using the combined species-gene symbol nomenclature including gene and exon of origin when relevant. Bead synthesis errors were assessed and when possible, altered unique molecular identifiers (UMIs) were repaired. Cell barcode abundance was tallied, and gene expression was called for the top 10,000 cell barcodes. Count matrices of genes x cells were imported in the R v. 3.6 statistical environment and Seurat (v 4.0.5) was used as the primary analytical package (Hao et al., 2021). Count matrices were merged into a single Seurat object, which was split by the sample of origin. Human, murine and *P. vivax* genes and transcripts counts were tallied for each cell barcode.

#### scRNA-seq analysis of *P. vivax* transcriptomes

The Seq-Well analytical pipeline was run on the samples as described above. For samples for which pre- and post-capture data were available (batch 2), matching cell barcodes were identified in the corresponding genes x cell matrices and UMI counts were summed over pre- and post-capture libraries. The resulting matrices were merged into a Seurat (v 4.0.5) object (2,171 cells, 85,276 genes). Only *P. vivax* genes were retained for subsequent analysis. Cell outliers were initially removed from the dataset by running the runColDataPCA() function in scater (v 1.20.1) (McCarthy et al., 2017). Samples with > 5% of mitochondrial genes and > 30% of total reads mapping to rRNA genes were also excluded from the analysis. Finally, the Seurat object was split by batch and each individual object was further filtered by excluding cells with fewer than 20 genes and 50 reads for batch 1 and with fewer than 100 genes and 200 reads for batch 2. The two objects were then normalized with SCTransform and integrated using the following functions in Seurat: SelectIntegrationFeatures(), PrepSCTIntegration(), FindIntegrationAnchors(), RunPCA(), IntegrateData(). Transcriptomes were visualized using UMAP dimensionality reduction in Seurat with n.neighbors = 20, min.dist = 0.3, repulsion.strength = 0.02, and local.connectivity = 3. For the identification of cluster-specific gene markers and differential gene expression between AP2G-positive and AP2G-negative cells, the FindAllMarkers() and FindMarkers() functions in Seurat were used with a MAST test.

#### Bulk RNA-seq analysis

fastq files were mapped using STAR v. 2.5.3a (Dobin et al., 2013) against PVP01 *P. vivax* v1 genome assembly and annotation, and quantitated by RSEM v. 1.3.0 (Li and Dewey, 2011). Differential expression analysis was performed in DESeq2 on protein-coding genes (Love et al., 2014).

#### Comparison between *P. vivax* single-cell and bulk transcriptomes

*P. vivax* single cells were assigned one of several possible developmental stages based on gene expression correlations between scRNA-seq and bulk transcriptomes. Pearson and Spearman correlations were computed using the Hmisc package in R (v 4.6-0).

#### Comparison between *P. vivax* and *P. berghei* single-cell transcriptomes

*P. vivax* cells were compared with a subset of the mouse Malaria Cell Atlas dataset (liver and blood asexual and sexual stages; https://zenodo.org/record/2843883#.Ygz7wC-l1bU) using the scmapCell() function in scmap (v 1.14.0) (Kiselev et al., 2018) with the MCA data as the reference index and the *P. vivax* data as the query dataset. Each cell was given a stage assignment based on the top matched cell from the reference index.

#### scRNA-seq analysis of human transcriptomes

Genes x cell data matrices were called for 10000 cells in each sample and merged in the R computational environment (R v. 3.6.0) using the Seurat v. 3.2.3 computational package. Cell barcodes had to meet the following conditions to be retained: displaying a minimum of 300 transcripts and 200 genes (across the three genomes), with less than 5 percent murine mitochondrial transcripts and less than 30 percent of human mitochondrial transcripts (to account for the mitochondrial richness of hepatocytes). Additionally, log10 ratios of human and murine-mapped transcripts per cell barcodes were calculated, and only cell barcodes with a ratio greater than 0 were retained. The resulting merged Seurat object was log-normalized to 10,000 transcripts (NormalizeData function), and variable features were selected using Seurat’s FindVariableFeatures() function, picking 2,000 genes based on the variance-stabilizing transform. The merged object was scaled and centered using a linear model, with scale.max=10, block.size =1,000 and min.cells.to.block=3,000. Principal component analysis was performed using RunPCA, retaining the top 12 components. The number of principal components retained was picked based on the inspection of the “elbow plot” and JackStraw procedure (n=100 replicates, 50 PCs) in Seurat. A UMAP embedding was calculated on the top 12 principal components using the RunUMAP() function with n.neighbors=50, min.dist = 0.3, spread=1, metric = "correlation". Cluster identification was based on the Louvain algorithm with the top 12 principal components and 20 nearest neighbors at a resolution of 0.3. Gene and transcript coverage plots per cell barcodes were generated on the merged dataset prior to the final filtering step (for barcodes with a 10-fold enrichment for human transcript). Cluster markers were identified using the FindAllMarkers() function with logfc.threshold set to 0.58, min.diff.pct = 0.20, and test.use = "MAST". For differential gene expression between infected and non-infected cells or between cells carrying AP2-G positive and AP2-G negative parasites the FindMarkers() function set to logfc.threshold = 0.25 and test.use = "MAST" was used. Functional class enrichment on differentially expressed gene sets was carried out using the Gene Ontology (GO) tool (Gene and Consortium, 2021; Gene et al., 2011).

#### Quantitative RT-PCR

Total RNA from pooled triplicate wells of *P. vivax*-infected MPCCs was extracted with TRIzol (Thermo Fisher), DNAse treated and purified using the RNeasy MinElute Cleanup Kit (Qiagen). cDNA synthesis was performed using SuperScript II (Thermo Fisher) and RT-PCR was carried out using PowerUp SYBR Green Master Mix (Applied Biosystems) in a Roche Light Cycler 480 Real-Time PCR Detection System according to the manufacturer’s instructions. The primers used are listed in Table S6. Relative gene expression was calculated with the delta-delta Ct method, using PVP01_1213400 as housekeeping gene.

#### Immunofluorescence analysis

*P. vivax*-infected MPCCs were fixed in ice-cold methanol or 4% paraformaldehyde (PFA), washed in phosphate-buffered saline (PBS) and stored at 4°C. Parasites were detected using *P. vivax*-specific antibodies (PvUIS4, PvBip and PvCSP) on methanol fixed cells as described in (Gural et al., 2018). For IFITM3 staining, PFA-fixed cells were permeabilized with 0.2% TritonX100 for 10 minutes at room temperature, washed in PBS and blocked with 2% bovine serum albumin (BSA) in PBS for 30 minutes at room temperature. IFITM3 rabbit monoclonal antibody (Cell Signaling) was incubated overnight at 4°C (1:100). Alexa-conjugated 488 secondary anti-rabbit antibody (1:1000) was incubated for 1 hour at room temperature, followed by nuclear staining with Hoechst. Images were captured on a Nikon Eclipse Ti or Zen-ApoTome inverted wide-field microscopes using 20x objectives. To quantify parasite size, the area of parasite defined by the PvUIS4 staining was measured using NIS-Elements Microscope Imaging Software and automatically converted to equivalent diameter.

#### Fluorescence *in situ* hybridization

*P. vivax*-infected MPCCs were fixed in 3.7% PFA for 10 minutes at room temperature, washed in PBS, immersed in 70% ethanol and stored at 4°C. Custom labelled probes set specific to *Pv18S rRNA* (FAM dye) and *PvGEXP5* (Quasar 670 dye) purchased from Stellaris were hybridized overnight in the dark at 37°C following the manufacturer’s instructions. After nuclear staining and washing, cells were imaged in a Nikon Eclipse Ti fluorescence microscope as described above. To quantify parasite size, the area of parasite defined by the *Pv18S rRNA* staining was measured using Fiji Software (Schneider et al., 2012) and converted to diameter.

### QUANTIFICATION AND STATISTICAL ANALYSIS

*n* represents the number wells from each plate as described in the figure legends. Exception for Figure 2C where *n* represents 2 independent infections. Methods used for computing statistical significance are indicated in figure legends. Statistical significance was considered for p values below 0.05. Data was analyzed using GraphPad Prism Software.

## Supplementary Material

Table S2 Table S2. Summary statistics for each parasite and host cell analyzed across the dataset. Related to Figures 1 to 3.

Table S5 Table S5. Summary statistics for differential gene expression analyses between the hepatocyte subsets. Related to Figure 4.

Table S4 Table S4. Summary statistics for differential gene expression and GO term enrichment analyses for the parasite subsets. Related to Figures 1 to 3.

Table S3 Table S3. *P. vivax* bulk RNA-seq dataset. Related to Figure 1.

Supplemental
