# Supplementary material for: A single-cell liver atlas of Plasmodium vivax infection
Source: Cell Host Microbe. Author manuscript; Available in PMC 2025 Jul 23. (PMC12285726; doi:10.1016/j.chom.2022.03.034)
Supplement: Supplemental [file NIHMS2083125-supplement-Supplemental.pdf]

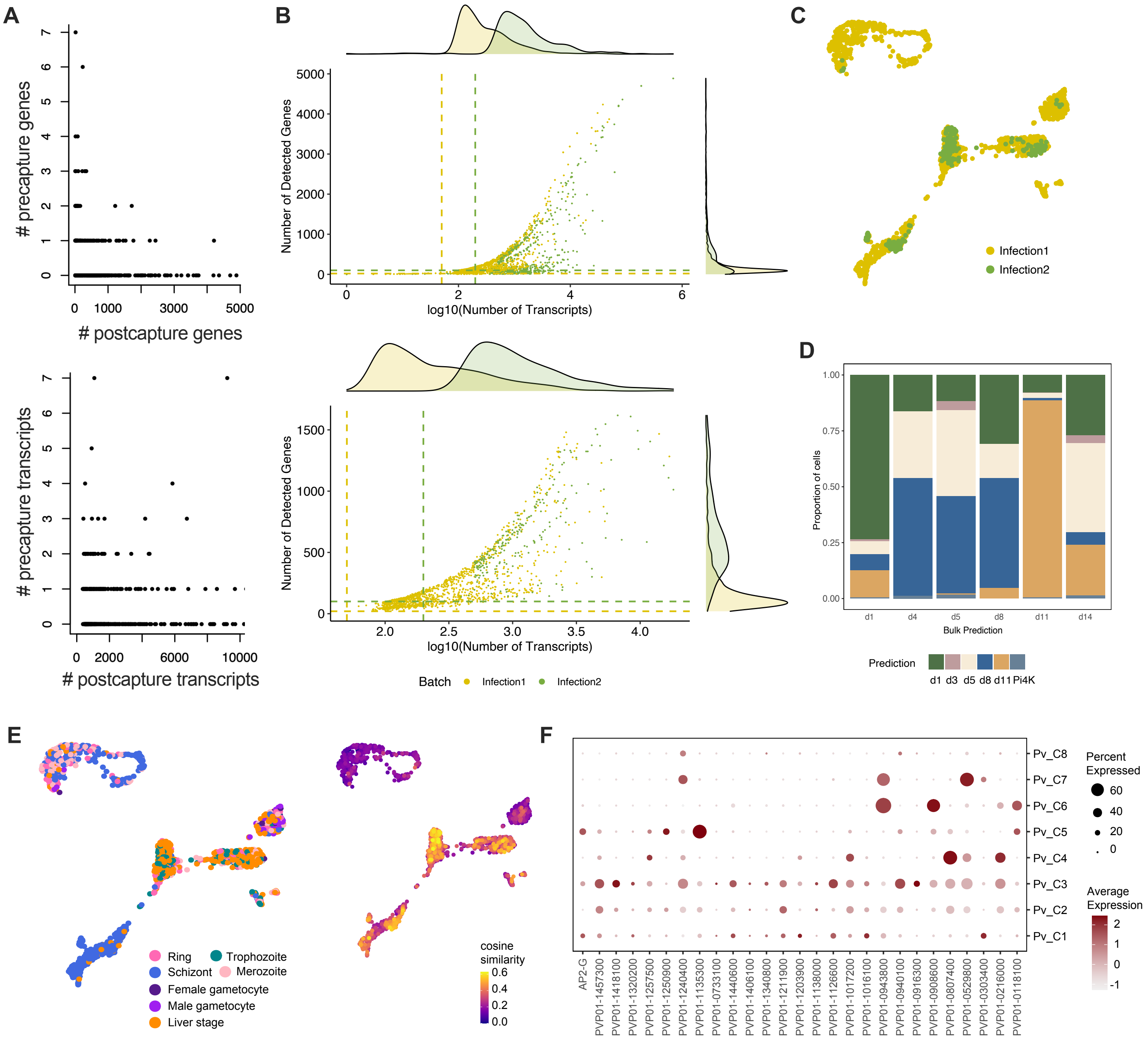

Figure S1. Quality control and comparison of *P. vivax* transcriptomes with bulk and single-cell reference data sets. Related to **Figure 1. A.** Pairwise comparisions of *P. vivax* gene (top) and trancript (bottom) numbers in pre- and post-capture conditions. **B.** Distributions of *P. vivax* gene and trancript numbers per batch in pre (top) and post (bottom) filtering. The lines indicate were cutoffs were applied to filter low quality cells in each batch. **C.** Distribution of infections 1 and 2 in the global UMAP. **D.** Proportion of cells by predicted stage based on spearman correlations with bulk data. **E.** Scmap of *P. vivax* transcriptomes to the *P. berghei* MCA dataset. Cells are coloured by matched cell type in the MCA dataset (left) or the cosine similarity (right). **F.** Dotplot showing the ApiAP2 expression profile across the different clusters.

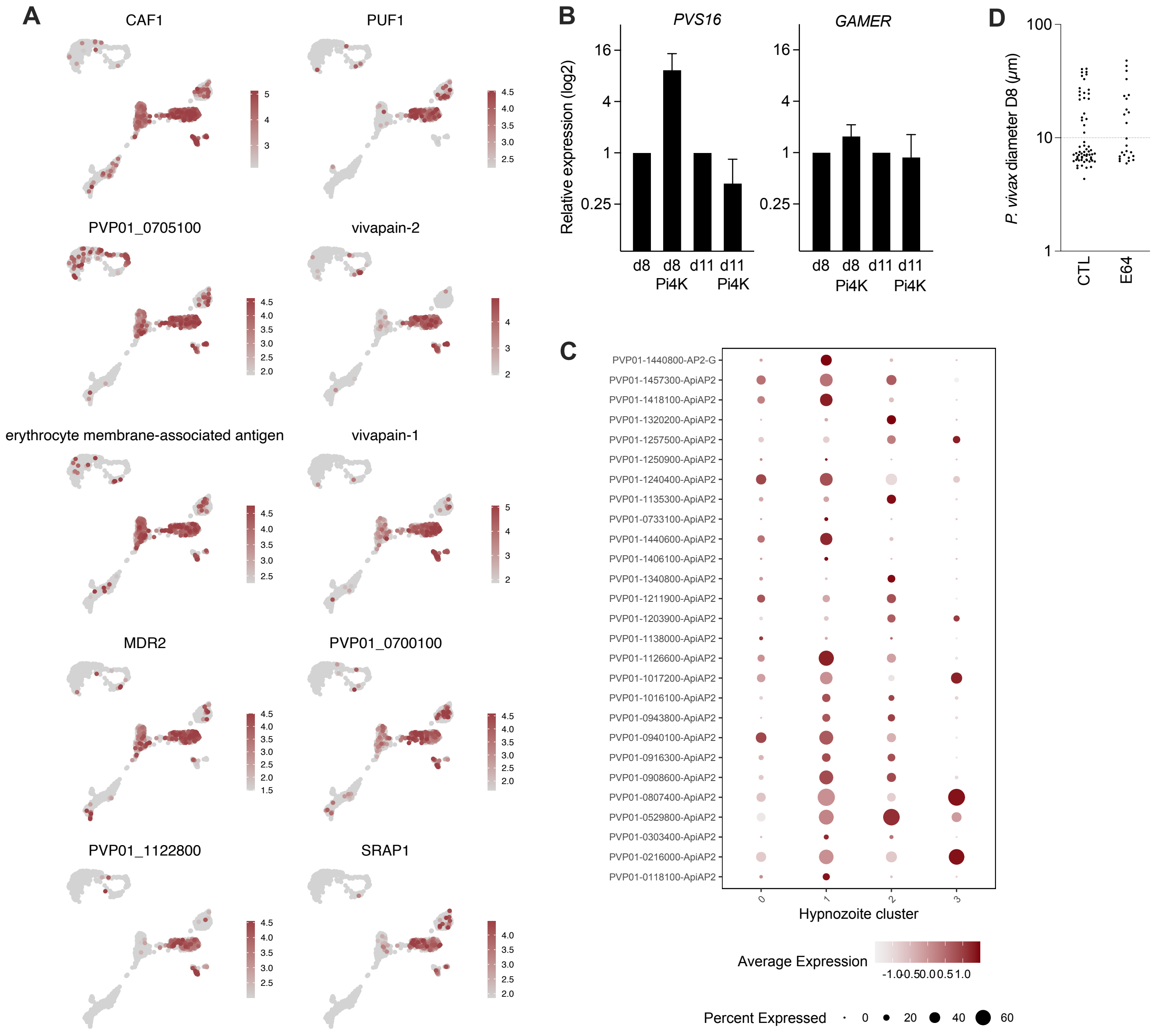

Figure S2. Hypnozoite-specific gene expression. Related to Figure 3. A. UMAP highlighting the expression of the 10 genes used to calculate the hypnozoite score. **B.** Quantitative RT-PCR analysis of gametocyte markers (*PVS16* and *GAMER*) in Pi4K-treated bulk samples on days 8 and 11. Non-treated samples were set to 1 (mean ± SEM; *n* = 2 independent infections). **C.** Dotplot showing scaled expression of the 27 members of the ApiAP2 family of transcription factors across the hypnozoite subclusters. **D.** Parasite size distribution in MPCC cultures treated with protease inhibitor E64 (1 *µ*M) for 8 days. Each dot represents an individual parasite (*n* = 3-5 wells pooled).

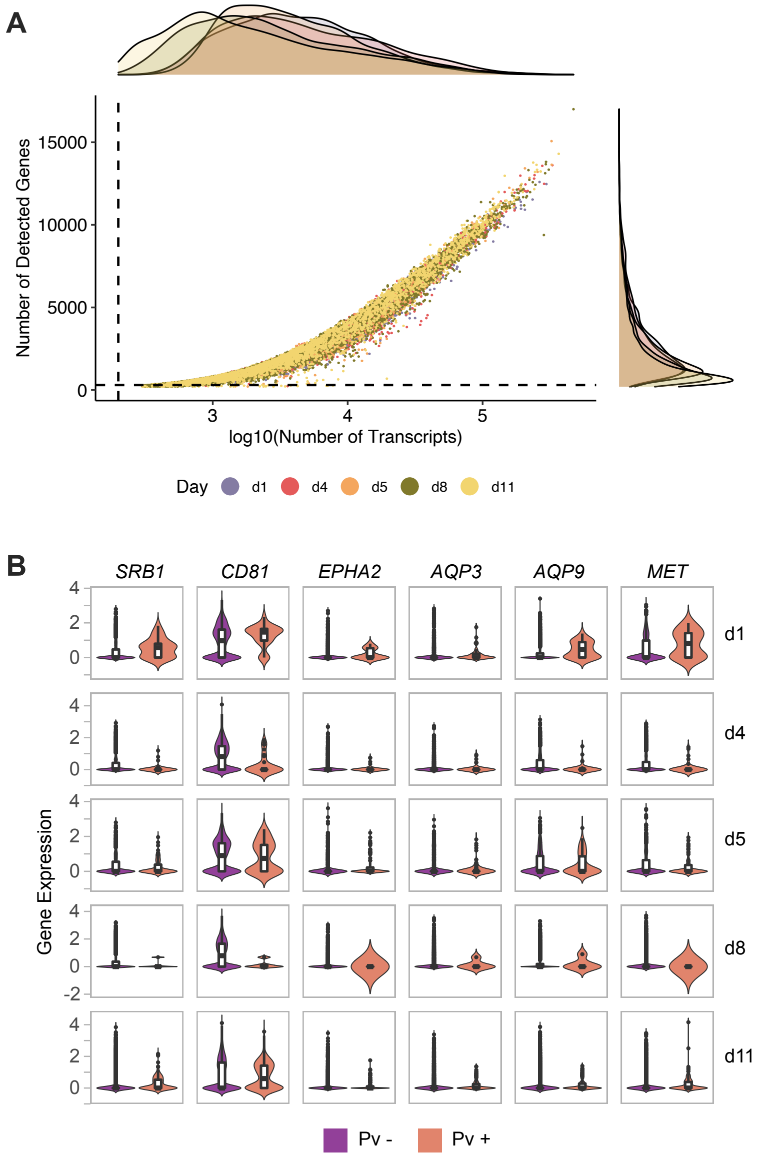

Figure S3. Quality control and expression of putative host-cell entry factors by infection status. Related to Figure 4. A. Quality controls for single-cell human transcriptomes of batch infection 2. The lines show the gene and transcript cutoffs applied to filter low quality cells. Samples are coloured by collection day. **B.** Violin plots showing expression of host factors with previously described roles in infection by other *Plasmodium*.

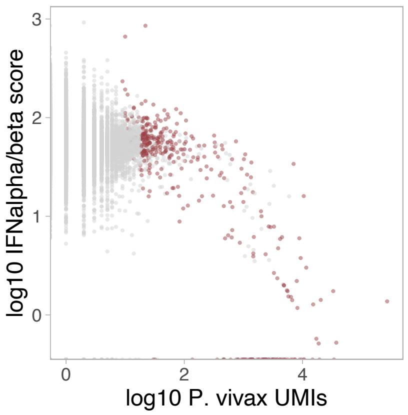

Figure S4. Interferon (IFN) response. Related to Figure 5. A. Scatter plot showing higher IFN response in *P. vivax*-negative cells (grey) acrross all clusters. *P. vivax*-positive hepatocytes are colored in red.

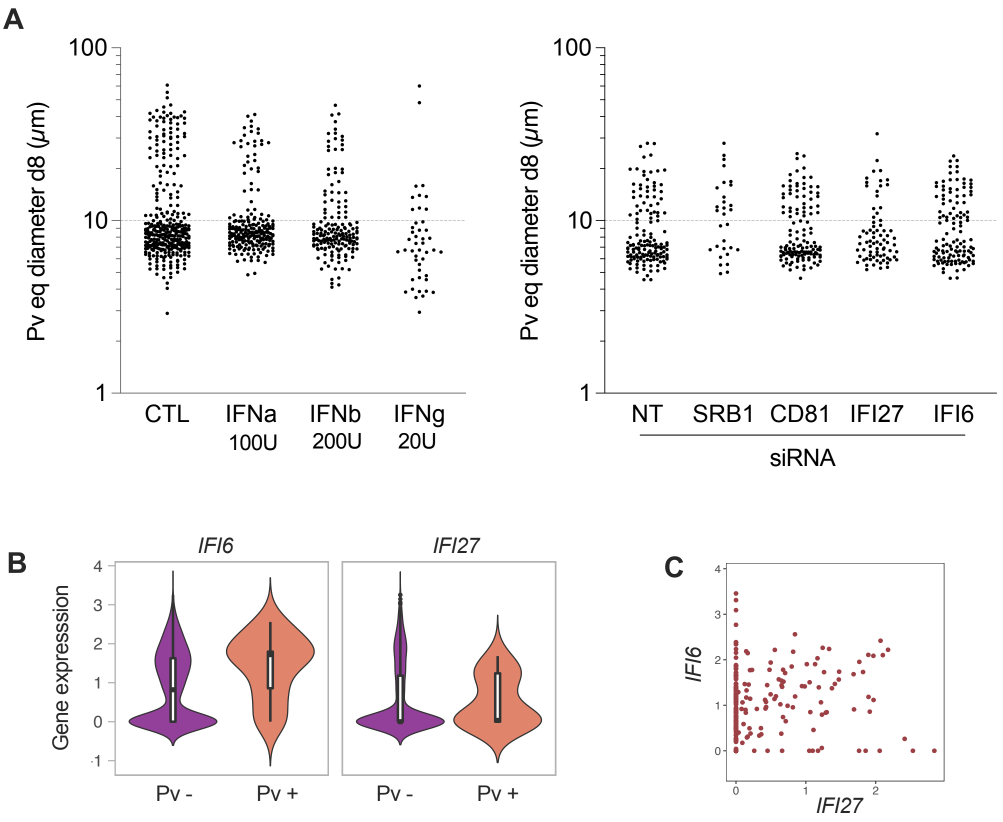

Figure S5. Effect of IFN and siRNA treatment on parasite development. Related to Figure 6. A. Scatter plot showing the parasite size distribution in MPCC cultures treated with interferon post infection (left) or siRNA prior infection (right). Interferon alpha (IFNa), beta (IFNb) and gamma (IFNg) were added on day 5 after infection for 3 days. Non-targeted (NT) siRNA control and targeting siRNA molecules were added to hepatocytes 3 days prior *P. vivax* infection. Cultures were fixed and analyzed on day 8. Each dot represents an individual parasite (*n* = 5-6 wells from 2 independent experiments pooled). The traced line indicates the 10 μm cutoff used for hypnozoite staging. **B.** Expression of *IFI6* and *IFI27* in uninfected (Pv-) and *P. vivax*-infected (Pv+) hepatocytes on day 1. **C.** Expression of *IFI6* and *IFI27* in *P. vivax*-positive hepatocytes.

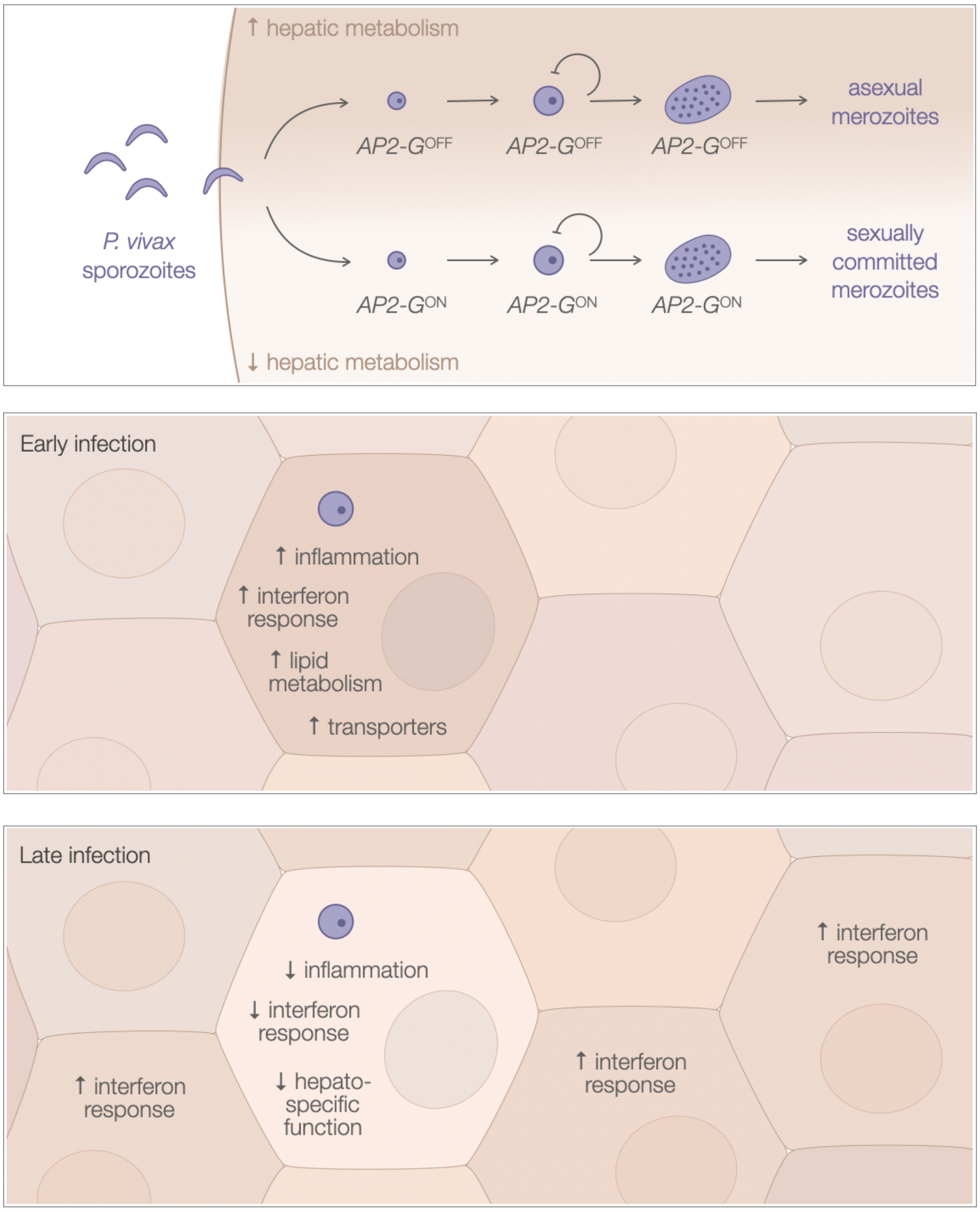

Figure S6. Model depicting the interaction between *P. vivax* and host cell transcriptional responses upon infection. Related to Figures 1 to 5. *P. vivax* parasites are colored in purple and hepatocytes in shades of brown to highlight cell heterogeneity.

Table S1. Quality control of single-cell *P. vivax* transcriptomes. Related to Figure 1.

Batch	Genes per cell cut off	Reads per cell cut off	Time post-infection	Treatment	# Passed QC	Median genes per cell	Total genes (Genome coverage %)
Infection 1	20	50	d1	Ctrl	207	148	5156 (75.2)
			d5	Ctrl	304	167	4600 (67)
			d11	Ctrl	526	92	4710 (68.6)
			d14	Ctrl	162	196.5	4251 (62)
			d14	Pi4K	71	259	3833 (55.9)
Infection 2	100	200	d4	Ctrl	80	604.5	4587 (66.9)
			d5	Ctrl	2	-	393 (5.7)
			d8	Ctrl	37	721	4051 (59)
			d8	Pi4K	28	582	3566 (52)
			d11	Ctrl	23	193	2623 (38.2)
			d11	Pi4K	54	536.5	4071 (59.3)

Table S6. Primers. Related to Figures 2 and 3.

Gene ID	Gene symbol	Primer forward	Primer reverse
PVP01_0115300	GAMER	ACGGATTTTTGCCACCAAGCGC	TCGTCCAATTTGATGGCACCACGC
PVP01_0305600	PVS16	TCCTCCTCTTGTGTGTCCTGCT	AAGCGTTTTCTGCATCAGGGGG
PVP01_1201400	GEXP5	ACGAAAAATCTTCCGCCGCGA	TTGGCCAGCACCTTGTTGTCCA
PVP01_1467200	G377	ACAACCCCAAGCTGCACGAGTT	AGCTCGGTCGCAAATTGGGGGTTT
PVP01_1213400	60S	ACGAATTTTGGATGCCGGGGGA	TTTCTTCCCTTGGAGCGGACGT
